# Dedication: Anne Heloise Theo (28 August 1985–6 February 2023)

**DOI:** 10.1098/rstb.2023.0041

**Published:** 2023-06-05

**Authors:** Kartik Shanker

**Affiliations:** ^1^ Centre for Ecological Sciences, Indian Institute of Science, C.V. Raman Avenue, Bangalore 560012, India; ^2^ Dakshin Foundation, No. 2203, 8th Main, D Block, MCECHS Layout, Sahakara Nagar, Bengaluru 560092, India


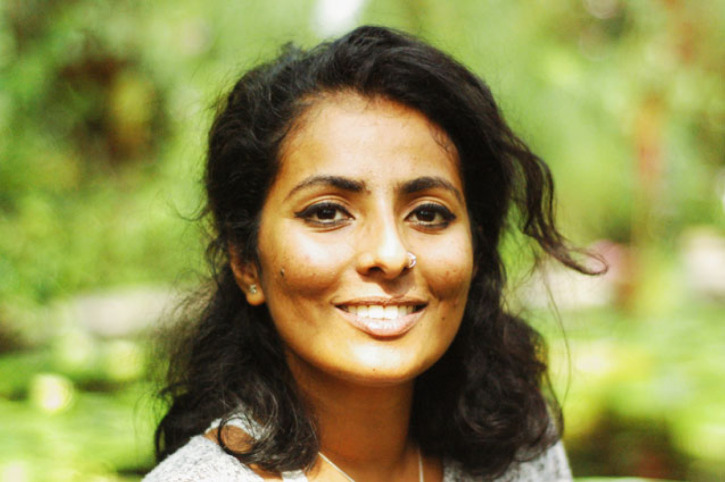


*This theme issue is dedicated to Anne Theo, one of the Guest Editors of this issue, who sadly passed away during the final stages of editing the issue. This dedication is written by Kartik Shanker, who was Anne's PhD advisor and co-author with her of a paper in this issue.*

One story captures Anne Theo's spirit and personality best. On her first work dive in the Lakshadweep Islands, Anne got separated from her group. Her fellow researchers and I surfaced after the mandatory search and became increasingly worried as there was no sign of her. The sea was getting choppy and we were running out of options. Anne surfaced seconds later, about 10 m away, looked at us and said ‘Where were *you* guys?’ Anne was never lost, the rest of the world was.

Anne had not considered in-water research when she began her PhD at the Indian Institute of Science, Bangalore. In fact, she was not even a particularly good swimmer at the time. But once the project was conceived, she trained herself rapidly in the Institute's swimming pool and got her dive certification as well. However, a larger problem loomed. Many marine biologists she consulted were not enthusiastic about her plan to study mixed species groups (MSGs) in reefs—they thought that reef fish MSGs were too ephemeral and might not be interesting. Anne was undeterred—she spent her first field season in the Lakshadweep Islands gathering evidence that MSGs were common, could be video-graphed and that there were a host of interesting ecological questions that one could address about them. Her work, which emphasized fundamental ecological and behavioural differences between shoaling and attendant fish groups, would inform theoretical frameworks that were developed for a global review.

Anne had a long history of friendships and partnerships in the field. Over a period of several years, her buddies—on land and in the water—included researchers from multiple different institutions. More recently, she mentored junior students doing marine ecology in both analysis and fieldwork, including identifying surgeonfish and parrotfish species that only she could tell apart. She was a whiz at R, and taught courses and conducted workshops in statistics and quantitative ecology. She played a key role in developing and proposing this special issue, and edited five of the manuscripts herein.

Anne's plans for the world were unfettered. She wanted to create a science cooperative that transcended the politics and pitfalls of academia. She wanted to end patriarchy and capitalism. Tragically, before she could do any of those things, Anne passed away on 6 February 2023. She leaves behind a vast community of close friends, colleagues and family that will miss both her fierce arguments and easy affection. She is survived by her husband and fellow ecologist, Guillaume Demare, her brother, Dennis, and her mother, Mary. The lasting impression she made on people and the legacy of her research will live on.

